# Effects of the stress hyperglycemia ratio on long-term mortality in patients with triple-vessel disease and acute coronary syndrome

**DOI:** 10.1186/s12933-024-02220-3

**Published:** 2024-04-25

**Authors:** Yu Zhang, Lei Guo, Hao Zhu, Lin Jiang, Lianjun Xu, Dong Wang, Yin Zhang, Xueyan Zhao, Kai Sun, Channa Zhang, Wei Zhao, Rutai Hui, Runlin Gao, Jizheng Wang, Jinqing Yuan, Yunlong Xia, Lei Song

**Affiliations:** 1 State Key Laboratory of Cardiovascular Disease, Cardiomyopathy Ward, National Clinical Research Center of Cardiovascular Diseases, Fuwai Hospital, National Center for Cardiovascular Diseases, Chinese Academy of Medical Sciences and Peking Union Medical College, 167, Beilishilu, Xicheng District, Beijing, 100037 People’s Republic of China; 2 Department of Cardiology, The First Affiliated Hospital of Dalian Medical University, 222, Zhongshan Road, Dalian City, 116011 People’s Republic of China; 3 Department of Cardiology, Fuwai Hospital, National Center for Cardiovascular Diseases, Chinese Academy of Medical Sciences and Peking Union Medical College, 167, Beilishilu, Xicheng District, Beijing, 100037 People’s Republic of China; 4 Cardiomyopathy Ward, Fuwai Hospital, National Center for Cardiovascular Diseases, Chinese Academy of Medical Sciences and Peking Union Medical College, 167, Beilishilu, Xicheng District, Beijing, 100037 People’s Republic of China; 5 Information Center, Fuwai Hospital, National Center for Cardiovascular Diseases, Chinese Academy of Medical Sciences and Peking Union Medical College, 167, Beilishilu, Xicheng District, Beijing, 100037 People’s Republic of China; 6 National Clinical Research Center of Cardiovascular Diseases, Fuwai Hospital, National Center for Cardiovascular Diseases, Chinese Academy of Medical Sciences and Peking Union Medical College, 167, Beilishilu, Xicheng District, Beijing, 100037 People’s Republic of China

**Keywords:** Acute coronary syndrome, Triple-vessel disease, Stress hyperglycemia, Cardiovascular mortality, Diabetes, Risk stratification

## Abstract

**Aims:**

Risk assessment for triple-vessel disease (TVD) remain challenging. Stress hyperglycemia represents the regulation of glucose metabolism in response to stress, and stress hyperglycemia ratio (SHR) is recently found to reflect true acute hyperglycemic status. This study aimed to evaluate the prognostic value of SHR and its role in risk stratification in TVD patients with acute coronary syndrome (ACS).

**Methods:**

A total of 3812 TVD patients with ACS with available baseline SHR measurement were enrolled from two independent centers. The endpoint was cardiovascular mortality. Cox regression was used to evaluate the association between SHR and cardiovascular mortality. The SYNTAX (Synergy Between Percutaneous Coronary Intervention With Taxus and Cardiac Surgery) II (SSII) was used as the reference model in the model improvement analysis.

**Results:**

During a median follow-up of 5.1 years, 219 (5.8%) TVD patients with ACS suffered cardiovascular mortality. TVD patients with ACS with high SHR had an increased risk of cardiovascular mortality after robust adjustment for confounding (high vs. median SHR: adjusted hazard ratio 1.809, 95% confidence interval 1.160–2.822, *P* = 0.009), which was fitted as a J-shaped pattern. The prognostic value of the SHR was found exclusively among patients with diabetes instead of those without diabetes. Moreover, addition of SHR improved the reclassification abilities of the SSII model for predicting cardiovascular mortality in TVD patients with ACS.

**Conclusions:**

The high level of SHR is associated with the long-term risk of cardiovascular mortality in TVD patients with ACS, and is confirmed to have incremental prediction value beyond standard SSII. Assessment of SHR may help to improve the risk stratification strategy in TVD patients who are under acute stress.

**Supplementary Information:**

The online version contains supplementary material available at 10.1186/s12933-024-02220-3.

## Introduction

Coronary artery disease (CAD) remains the leading cause of death worldwide [1]. Triple-vessel disease (TVD) is a severe type of CAD that impacts the blood supply of all three major coronary arteries (left anterior descending, left circumflex, and right coronary). TVD is estimated to be present in approximately 30% of patients with CAD, with a substantial risk of mortality that requires timely risk stratification [2]. The SYNTAX (Synergy Between Percutaneous Coronary Intervention With Taxus and Cardiac Surgery) II (SSII) score has been developed to predict long-term mortality in patients with TVD but has only moderate discrimination ability [3]. 

A substantial number of clinical studies have identified that abnormal glucose metabolism is highly prevalent among patients with CAD and is also involved in the progression of CAD [4–6]. Stress hyperglycemia refers to a transient increase in blood glucose at the time of admission and represents the regulation of glucose metabolism by the organism in response to stress [7]. Classical evaluation of stress hyperglycemia relies on glucose concentrations at admission and is markedly affected by background glycemia. The stress hyperglycemia ratio (SHR), which uses the estimated average glucose derived from glycated hemoglobin (HbA_1c_), is now proposed to more effectively reflect the acute hyperglycemic state [8]. 

Previous studies have demonstrated the prognostic value of the SHR in patients with acute coronary syndrome (ACS) [9–11]. However, there have been no evaluations of the associations of SHR with outcomes in TVD patients who present ACS. A recent study indicated that a higher SHR was significantly correlated with an increased risk of multi-vessel involvement among patients with CAD, emphasizing the distinct role of SHR in TVD [12]. 

This study investigated the association of SHR with long-term cardiovascular mortality in a large multicenter cohort of TVD patients with ACS and evaluated whether addition of the SHR could improve the performance of the currently established SSII model in terms of prediction of cardiovascular mortality.

## Subjects, materials and methods

### Study design and population

**Fig. 1:**
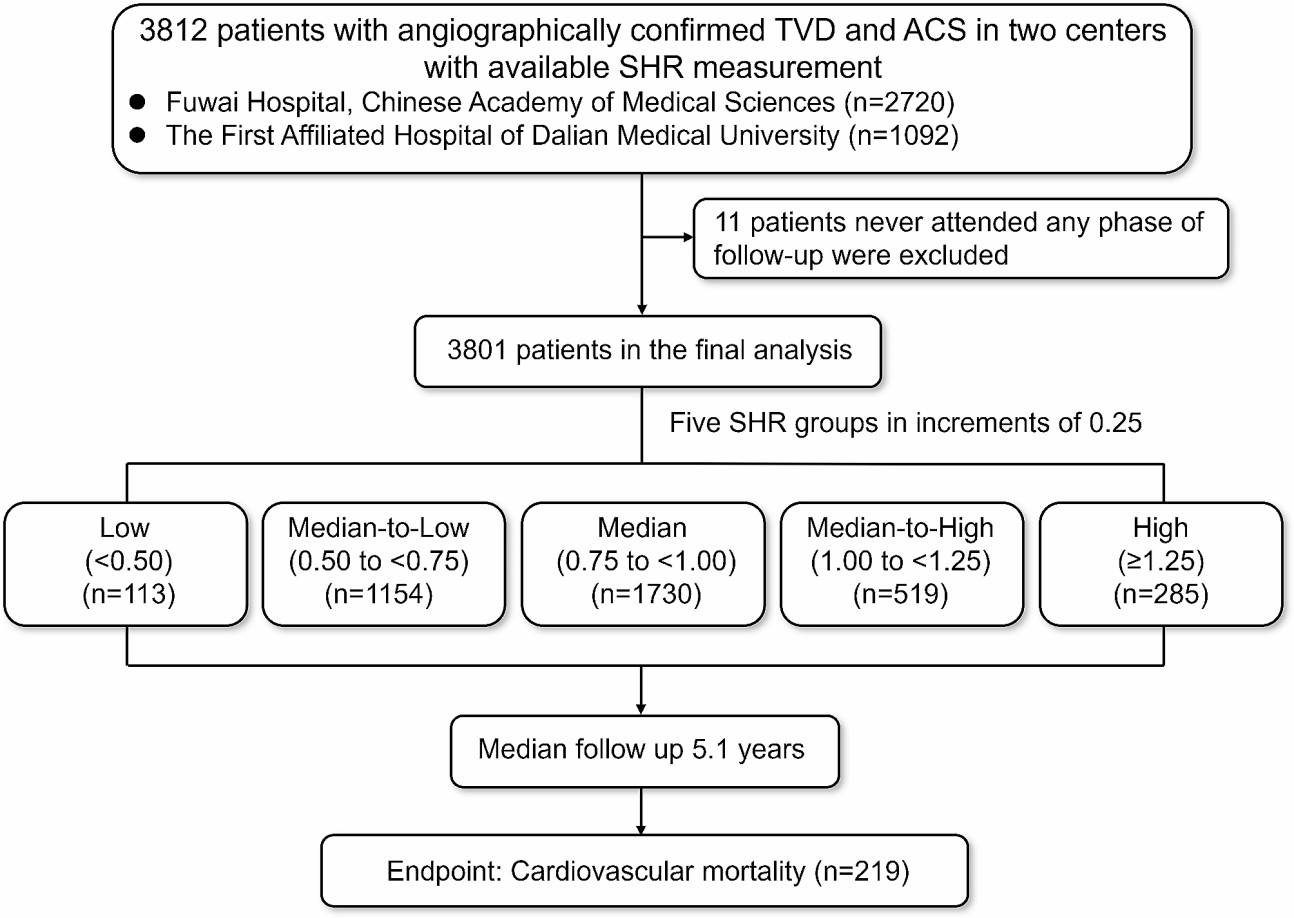
Flowchart of this study. ACS, acute coronary syndrome; SHR, stress hyperglycemia ratio; TVD, triple-vessel disease

The study had a multicenter cohort design and prospectively recruited 3812 TVD patients presenting ACS and having available admission blood glucose and HbA_1c_ measurements at Fuwai Hospital, Chinese Academy of Medical Sciences and The First Affiliated Hospital of Dalian Medical University. Patients at Fuwai Hospital (*n* = 2720) were recruited between 2004 and 2011 and those at The First Affiliated Hospital of Dalian Medical University (*n* = 1092) were recruited between 2013 and 2018. All patients studied were recruited at admission. TVD was defined as angiographically confirmed stenosis of ≥ 50% in all three main epicardial coronary arteries (left anterior descending, left circumflex, and right coronary) with or without involvement of the left main artery. All patients underwent a detailed clinical examination and coronary artery bypass grafting (CABG), percutaneous coronary intervention (PCI), or medical therapy alone in accordance with the current practice guidelines, judgment of the heart team, and patient preference. The presence of diabetes was defined as a previous diagnosis of diabetes and an HbA_1c_ ≥ 6.5%. Patients who never attended any phase of follow-up (*n* = 11) were excluded, leaving data for 3801 patients available for final analysis. The flow chart showing the patient selection process is provided in Fig. 1.

The study was approved by the Ethics Committee of Fuwai Hospital and The First Affiliated Hospital of Dalian Medical University and performed in accordance with the principles of the Declaration of Helsinki. Informed consent was obtained from all study participants.

### Measurement and calculation of the SHR

Venous blood samples were collected from the enrolled patients in serum separator tubes by direct venipuncture within 24 h after admission. The levels of blood glucose and HbA_1c_ on admission were measured using standard biochemistry techniques in the core laboratory at Fuwai Hospital and the first affiliated hospital of Dalian Medical University. The SHR was calculated as follows:

admission blood glucose (mmol/L)/[1.59 × HbA_1c_ (%) − 2.59].

### Follow-up and outcomes

Patients recruited from Fuwai Hospital were followed up annually until 2016, and those recruited from the first affiliated hospital of Dalian Medical University were followed up annually until 2022. Outcomes data were obtained by telephone interview, follow-up letter, or clinic visit. All events were carefully checked and verified by an independent group of clinical physicians. Investigator training, blinded questionnaire completion, and telephone recording were implemented to obtain high-quality data. The endpoint of the study was cardiovascular mortality.

### Statistical analysis

Continuous data are shown as the mean ± standard deviation or as the median [interquartile range]. Categorical variables are summarized as the percentage. Continuous variables were compared between groups using one-way analysis of variance and categorical variables using the chi-squared test.

For survival analyses, univariable and multivariable Cox proportional hazard regression models were used to evaluate the associations between various SHR values and cardiovascular mortality in patients with TVD with calculation of hazard ratios (HRs) and 95% confidence intervals (CIs). The multivariable Cox model was adjusted for age, sex, body mass index, previous myocardial infarction, previous stroke, hypertension, diabetes, chronic obstructive pulmonary disease, peripheral artery disease, chronic kidney disease, smoking, left ventricular ejection fraction, triglycerides, total cholesterol, low-density lipoprotein, high-density lipoprotein, left main involvement, and treatment strategies. When fitting Cox models, the reference group was chosen as the group with the lowest incidence of cardiovascular mortality (Additional file 1: Table S1). A restricted cubic spline analysis was performed using the *rms* package (version 6.7-1) to visualize the association of the SHR level with cardiovascular mortality. The restricted cubic spline analysis was also adjusted for above-mentioned variables.

The SSII model was used as the reference in the model improvement analysis. Receiver-operating characteristic curves were generated and C-statistics were compared for determining the added prognostic value of the SHR beyond the SSII. Weighted net reclassification improvement (NRI), as well as the event and non-event NRI were calculated using the *survNRI* package (version 0.1). All statistical analyses were performed using R software (v. 4.2.3, R Foundation for Statistical Computing, Vienna, Austria). A two-sided P-value of < 0.05 was considered statistically significant.

## Results

### Baseline characteristics

**Fig. 2:**
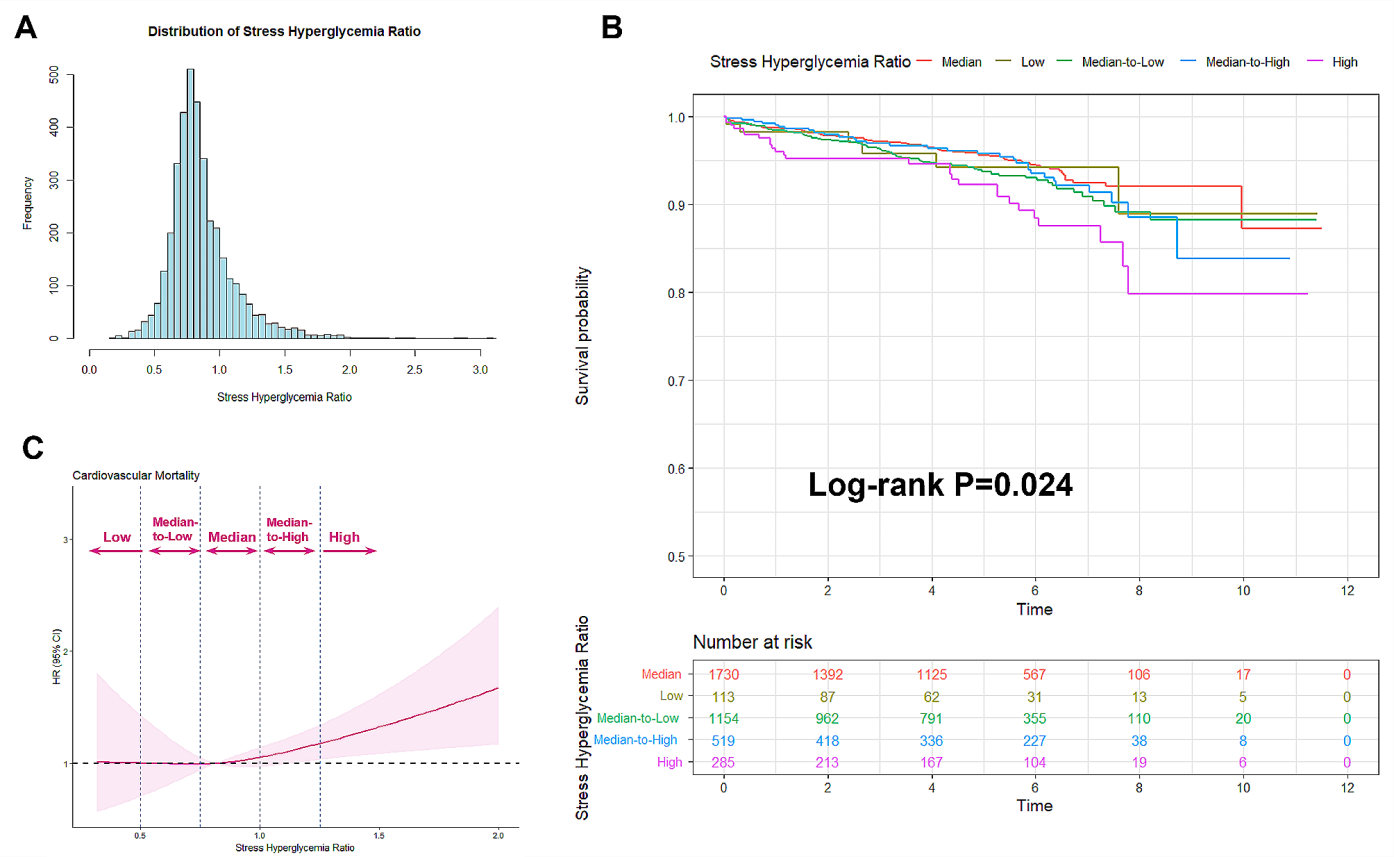
The association between SHR and cardiovascular mortality. (**A**) The distribution of the study population according to the SHR level; (**B**) The Kaplan–Meier survival curve showed the significant variation in survival according to SHR level; (**C**) The J-shaped association between SHR and cardiovascular mortality. CI, confidence interval; HR, hazard ratio; SHR, stress hyperglycemia ratio

The study population consisted of 3801 TVD patients with ACS. The mean age was 61.9 ± 10.3 years and 75.6% of the patients were male. The baseline information is summarized in Table 1. The distribution of the study population according to the SHR level is shown in Fig. 2A. The study participants were stratified into the following five SHR groups in increments of 0.25: low SHR (SHR < 0.50, *n* = 113), median-to-low SHR (SHR 0.50 to < 0.75, *n* = 1154), median SHR (0.75 to < 1.00, *n* = 1730), median-to-high SHR (1.00 to < 1.25, *n* = 519), and high SHR (SHR ≥ 1.25, *n* = 285). Both patients with lower and higher SHRs were associated with a higher incidence of diabetes, compared with patients with median SHR (*P* < 0.001).

**Table 1:** Baseline characteristics of the study population

	SHR groups					
	Low	Media-to-Low	Median	Median-to-High	High	P-value
	n = 113	n = 1154	n = 1730	n = 519	n = 285	
Age (years)	63.0 ± 9.5	62.2 ± 10.0	61.7 ± 10.5	61.8 ± 10.5	62.5 ± 9.8	0.337
Male	80 (70.8)	871 (75.5)	1324 (76.5)	394 (75.9)	204 (71.6)	0.313
BMI (kg/m^2^)	26.5 ± 2.5	26.0 ± 2.8	26.0 ± 2.5	26.0 ± 2.6	26.0 ± 2.6	0.504
Previous myocardial infarction	28 (24.8)	304 (26.3)	403 (23.3)	115 (22.2)	49 (17.2)	0.017
Previous stroke	10 (8.8)	127 (11.0)	177 (10.2)	71 (13.7)	47 (16.5)	0.010
Diabetes	113 (100.0)	630 (54.6)	792 (45.8)	269 (51.8)	145 (50.9)	< 0.001
Hypertension	75 (66.4)	800 (69.3)	1168 (67.5)	339 (65.3)	206 (72.3)	0.252
COPD	0 (0.0)	16 (1.4)	18 (1.0)	6 (1.2)	2 (0.7)	0.627
PAD	8 (7.1)	110 (9.5)	164 (9.5)	30 (5.8)	9 (3.2)	0.001
CKD	13 (11.5)	34 (2.9)	55 (3.2)	24 (4.6)	22 (7.7)	< 0.001
Current/former smoker	41 (36.3)	604 (52.3)	873 (50.5)	255 (49.1)	144 (50.5)	0.026
LVEF (%)	54.0 ± 9.8	56.9 ± 9.3	56.5 ± 9.2	55.9 ± 9.0	54.0 ± 10.2	< 0.001
Left main involvement	24 (21.2)	231 (20.0)	345 (19.9)	82 (15.8)	53 (18.6)	0.260
Admission blood glucose (mmol/L)	4.9 [4.1–5.7]	5.2 [4.7–6.2]	5.9 [5.3–7.4]	8.1 [6.3–10.6]	11.3 [8.3–15.3]	< 0.001
HbA_1c_ (%)	9.0 [7.6–10.7]	6.4 [5.9–7.6]	6.0 [5.6-7.0]	6.2 [5.3–7.6]	6.2 [5.3–7.8]	< 0.001
Triglycerides (mmol/L)	1.3 [1.1–1.9]	1.6 [1.2–2.1]	1.6 [1.2–2.2]	1.6 [1.2–2.2]	1.5 [1.1–2.2]	< 0.001
Total cholesterol (mmol/L)	4.3 [3.7–5.2]	4.6 [3.8–5.3]	4.6 [3.9–5.4]	4.6 [4.0-5.4]	4.6 [3.9–5.5]	0.022
LDL-C (mmol/L)	2.4 [1.9–3.1]	2.5 [2.0-3.1]	2.6 [2.1–3.2]	2.6 [2.1–3.3]	2.5 [2.1–3.2]	0.020
HDL-C (mmol/L)	1.0 [0.9–1.3]	1.0 [0.9–1.2]	1.0 [0.9–1.2]	1.0 [0.9–1.2]	1.0 [0.9–1.2]	0.540
Treatment strategy						
PCI	68 (60.2)	597 (51.7)	947 (54.7)	314 (60.5)	173 (60.7)	0.006
CABG	20 (17.7)	274 (23.7)	394 (22.8)	87 (16.8)	48 (16.8)	
MT	25 (22.1)	283 (24.5)	389 (22.5)	118 (22.7)	64 (22.5)	

Values are presented as mean ± standard deviation, median [interquartile range] or number (%)

BMI, body mass index; CABG, coronary artery bypass grafting; CKD, chronic kidney disease; COPD, chronic obstructive pulmonary disease; HbA_1c_, glycated hemoglobin; HDL-C, high-density lipoprotein cholesterol; LDL-C, low-density lipoprotein cholesterol; LVEF, left ventricular ejection fraction; MT, medical therapy; PAD, peripheral artery disease; PCI, percutaneous coronary intervention; SHR, stress hyperglycemia ratio

### Associations between SHR level and cardiovascular mortality in TVD patients with ACS

The median follow-up duration was 5.1 years [interquartile range 2.6, 6.5]. During follow-up, 219 patients died from cardiovascular causes, giving a cardiovascular mortality rate of 5.8%. Kaplan–Meier analysis showed that there was significant variation in survival according to SHR level (*P* = 0.024, log-rank test, Fig. 2B), with the lowest incidence of cardiovascular mortality in patients with the median SHR (Additional file 1: Table S1). Multivariable Cox regression analysis showed that patients with high SHR had an increased risk of cardiovascular mortality in comparison with the median SHR, after robust adjustment for confounding (patients with high SHR vs. median SHR: unadjusted HR 2.049, 95% CI 1.325–3.168, *P* = 0.001; adjusted HR 1.809, 95% CI 1.160–2.822, *P* = 0.009) (Table 2, Additional file 2: Table S2). Restricted cubic spline analysis visualized the associations between cardiovascular mortality and the SHR, showing a J-shaped pattern after confounding adjustment (Fig. 2C).

**Table 2:** Univariable and multivariable analysis of the association between SHR and cardiovascular mortality

SHR groups	Unadjusted HR (95% CI)	Unadjusted P-value	Adjusted HR (95% CI)	Adjusted P-value
Low	1.195 (0.521–2.740)	0.674	0.787 (0.337–1.840)	0.581
Median-to-Low	1.325 (0.966–1.816)	0.080	1.173 (0.852–1.615)	0.327
Median	Reference	Reference	Reference	Reference
Median-to-High	1.168 (0.771–1.767)	0.464	1.011 (0.666–1.534)	0.960
High	2.049 (1.325–3.168)	0.001	1.809 (1.160–2.822)	0.009

CI, confidence interval; HR, hazard ratio; SHR, stress hyperglycemia ratio

The detailed Cox models are present in Additional file 2: Table S2

### Differences in prognostic patterns of SHR between patients with and without diabetes

**Fig. 3:**
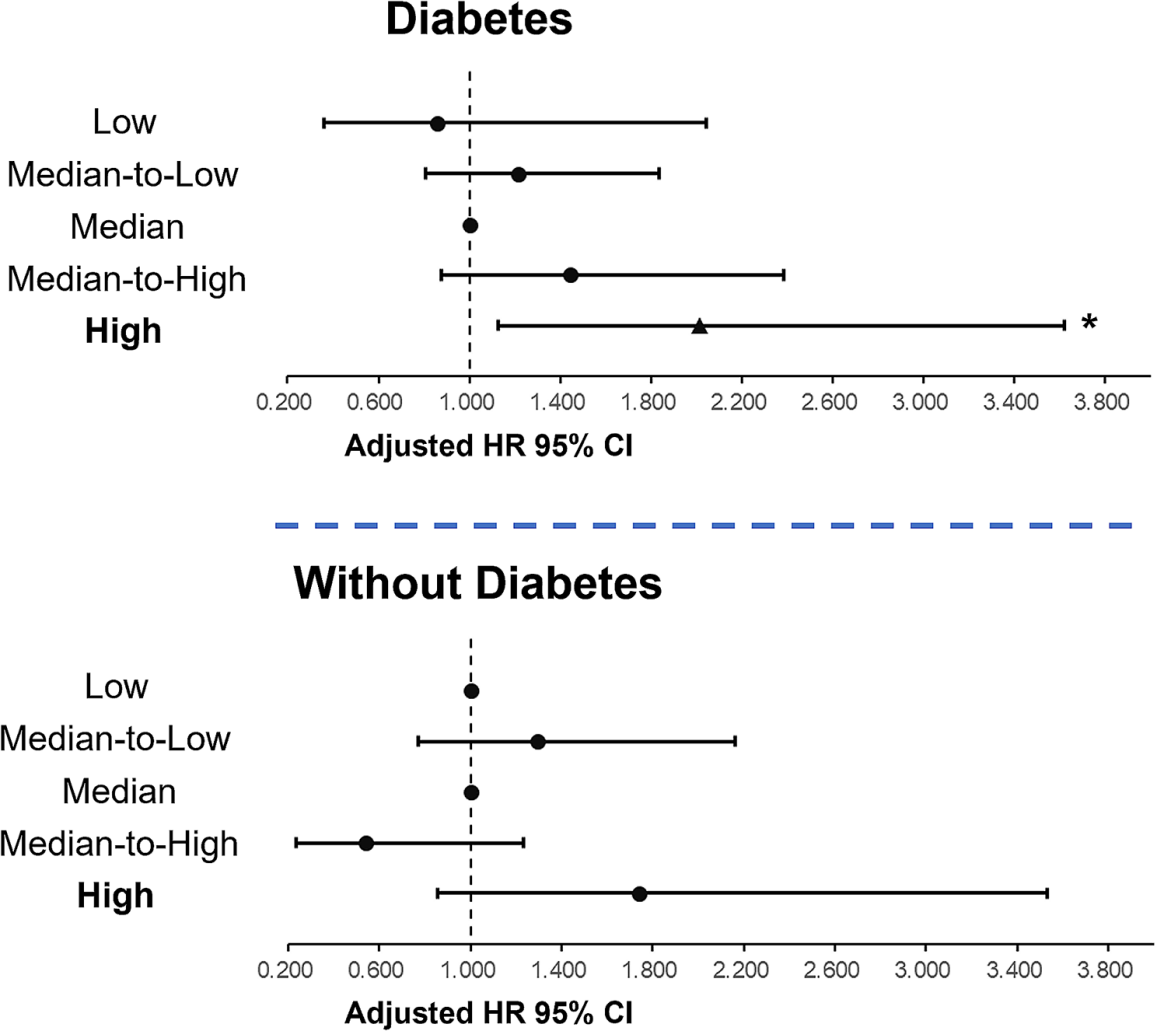
Different prognostic patterns of SHR between patients with and without diabetes. CI, confidence interval; HR, hazard ratio; SHR, stress hyperglycemia ratio. *indicates *P* < 0.05

Subgroup analyses were conducted according to whether or not diabetes was present. The results showed that the association between the SHR level and cardiovascular mortality was only significant in patients with diabetes and blunted in those without diabetes. (Fig. 3, Additional file 3: Table S3).

### Addition of SHR improves the SSII model

**Fig. 4:**
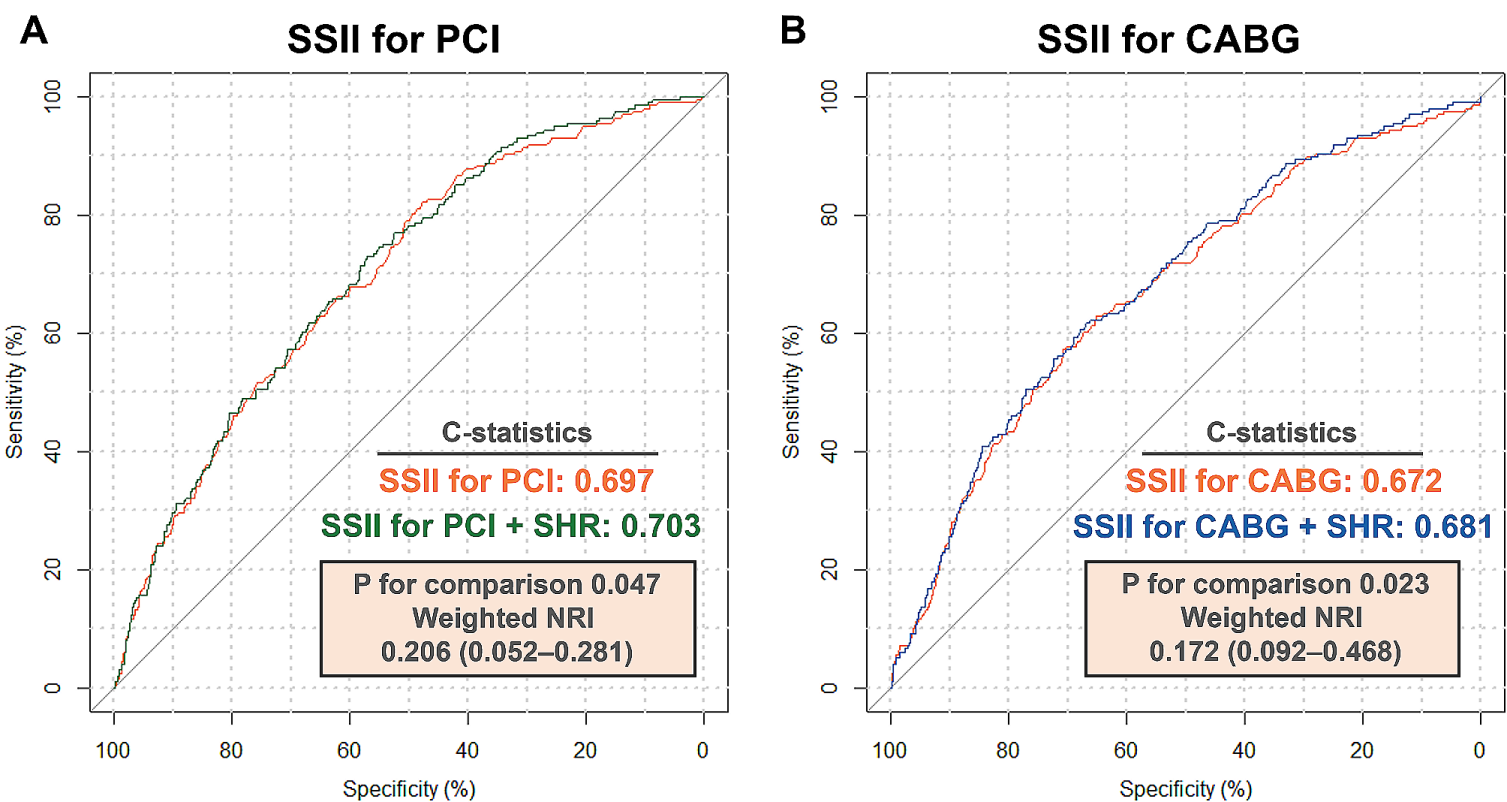
Addition of SHR improves the SSII model. (**A**) Addition of SHR improves the SSII model for PCI; (**B**) Addition of SHR improves the SSII model for CABG. CABG, coronary artery bypass grafting; NRI, net reclassification improvement; PCI, percutaneous coronary intervention; SHR, stress hyperglycemia ratio; SSII, Synergy Between Percutaneous Coronary Intervention With Taxus and Cardiac Surgery II

The SSII has become established as a tool for prediction of mortality in the long term in patients with TVD. The calculation of SSII was available in 2606 patients. The C-statistics of the original SSII for predicting cardiovascular mortality were 0.697 for PCI and 0.672 for CABG in TVD patients with ACS. When the SHR was added, the combined model achieved a higher C-statistic than did the original model (added to SSII for PCI: 0.703 vs. 0.697, *P* = 0.047; added to SSII for CABG: 0.681 vs. 0.672, *P* = 0.023). Moreover, SHR significantly improved the reclassification of the SSII (adding SSII for PCI: weighted NRI 0.206, 95% CI [0.052–0.281]; event NRI 0.223, 95% CI [0.078–0.293], non-event NRI -0.017, 95% CI [-0.041–0.030]; adding SSII for CABG: weighted NRI 0.172, 95% CI [0.092–0.468]; event NRI 0.194, 95% CI [0.122–0.397], non-event NRI -0.022, 95% CI [-0.037–0.288]) (Fig. 4).

## Discussion

This study investigated the association between various SHR levels and the risk of cardiovascular mortality in a multicenter cohort of TVD patients with ACS. Our results showed that high level of SHR was associated with an increased risk of long-term cardiovascular mortality in these patients. Moreover, we found that the prognostic value of SHR was more pronounced in patients with diabetes, but was blunted in those without diabetes. Inclusion of the SHR in the SSII model significantly improved the ability of the model to predict cardiovascular mortality.

Moderate hyperglycemia is an adaptive and protective process in response to acute illness, which increases the cellular uptake of glucose, thereby protecting energy metabolism in the myocardium under hypoxic or ischemic stress [13–15]. This corresponds to the fact that patients with median SHR had the lowest rate of cardiovascular mortality. However, an excessive stress hyperglycemia response is the presenting manifestation of impaired glycemic regulation. Development of stress hyperglycemia in patients with ACS is thought to be mediated by a complex interplay of disease-induced lipotoxicity, production of cytokines, and hormonal derangements [7, 16, 17]. Upon activation of the neurohormonal response, hepatic output of glucose increases via excessive gluconeogenesis, and insulin resistance can also be induced to exacerbate hyperglycemia [18, 19]. 

The association between stress-induced glucose concentrations and mortality in patients with ACS or acute myocardial infarction has been investigated previously [20, 21]. However, glucose concentrations at admission could be substantially impacted by the baseline glycemia of patients and thus cannot effectively reflect the actual stress response state. The SHR, which corrects glucose for HbA_1c_, is a recently introduced indicator [8] that controls the level of background glycemia and is a better biomarker than elevated admission glucose alone for identification of stress hyperglycemia [8, 22, 23]. A number of studies have demonstrated the SHR to have prognostic value in patients with several types of ACS [9–11], but its prognostic usefulness in TVD patients with ACS remains unclear. Degree of stress hyperglycemia is closely related to the severity of the disease [7, 12]. As one of the most severe forms of CAD, TVD triggers serious inflammation infiltration and endothelial dysfunction, which leads to more severely disordered glucose and lipid metabolism [24–26]. Our study is the first to indicate that the SHR had fair long-term prognostic ability in TVD patients with ACS, especially in those with diabetes.

The association between the SHR and cardiovascular mortality showed a J-shaped pattern. Patients with a high degree of stress hyperglycemia had a higher cumulative risk of cardiovascular mortality. It was possibly because strong glucose fluctuations and an excessive neurohormonal response caused by stress hyperglycemia in TVD patients with ACS triggered oxidative stress and aggravated vascular endothelial damage in multiple pre-existing lesions [7, 27, 28]. In addition, high perioperative stress hyperglycemia was also an important risk factor for the higher cardiovascular mortality in patients with ACS who underwent cardiac surgery [29, 30]. More importantly, we found that SHR documented at admission could predict poor long-term outcomes in TVD patients with ACS. There were two explanations. First, patients with mild or moderate stress hyperglycemia had the capability to maintain the metabolic homeostasis. Their elevation of glucose served as an adaptive response to the acute stress [14]. On contrast, the high level of SHR documented at enrollment indicated that the body was unable to effectively regulate the concentration of blood glucose. That is to say, patients with high SHR were more likely to develop metabolic alterations when countering a stress not only at this admission, but also during the period of follow-up, which eventually led to the adverse outcomes. Secondly, the high baseline SHR could cause glucose toxicity and trigger severe oxidative stress [31, 32], which might result in profound and irreversible damage to the coronary arteries [33], suggesting the causal relationship between baseline SHR and long-term prognosis.

The following subgroup analyses showed that the prognostic value of the SHR was found exclusively among patients with diabetes, which is consistent with previous reports [9, 34]. Fluctuations in blood glucose in patients with diabetes had a greater impact on the prognosis because they always had insulin resistance, which promotes glucotoxicity, lipotoxicity, and inflammation [7, 26]. In contrast, patients without diabetes might have had a generally more moderate level of stress hyperglycemia as seen in the baseline data, suggesting that their tolerance to stress was higher than that in patients with diabetes.

Accurate identification of patients with TVD at high risk of mortality can help to inform effective clinical management. In this regard, the SSII model was designed to predict mortality based on coronary anatomic complexity and several clinical variables [35]. We have shown that adding the SHR improves the performance of the SSII model, with significant reclassification. This finding indicates that the SHR can provide additional prognostic information beyond the SSII, suggesting that it is an important addition to risk stratification among TVD patients with ACS.

In conclusion, we have demonstrated a J-shaped association between the SHR and the risk of cardiovascular mortality in TVD patients with ACS, especially those with existing diabetes. Moreover, the SHR has been confirmed to have incremental value in predicting cardiovascular mortality over the standard SSII model and may help to optimize decision-making between PCI and CABG.

There were several limitations in this study. First was the lack of data regarding recovery of the SHR in this study. Further investigations on the association between the trajectory of the SHR and outcomes are warranted. Second, information related to the therapy carried out during follow-up was not available in this observational study, and patients were not followed centrally to guarantee the therapy optimization. Further clinical trial designs are needed to ensure the patients’ adherence to therapy and the therapy optimization during follow-up.

### Electronic supplementary material

Below is the link to the electronic supplementary material.

**Additional file 1: Table S1**. The incidences of cardiovascular mortality in five SHR groups.

**Additional file 2: Table S2**. Details of the univariable and multivariable analysis of the association between SHR and cardiovascular mortality.

**Additional file 3: Table S3**. Subgroup analysis of the association between SHR and cardiovascular mortality.

## Data Availability

The datasets used/or analyzed during the current study are available from the corresponding author on reasonable request.
